# Comparing the Force–Time Characteristics Between Countermovement and Assisted Countermovement Jump with Different Landing Strategies

**DOI:** 10.3390/muscles4040062

**Published:** 2025-12-17

**Authors:** Regine Y. S. Zhou, Lachlan P. James, Danny Lum

**Affiliations:** 1 High Performance Sport Institute, Singapore 397630, Singapore; regine_zhou@nysi.org.sg; 2 Sport Performance and Nutrition Research Group, School of Allied Health, Human Service and Sport, La Trobe University, Melbourne, VIC 3086, Australia; l.james@latrobe.edu.au

**Keywords:** landing impact force, jump height, propulsion duration, peak propulsion force

## Abstract

Researchers comparing countermovement (CMJ) and assisted countermovement (ACMJ) jumps reported conflicting findings on the landing impact force (LIF). This was likely due to differences in the landing strategies used. As the magnitude of LIF may have implications on neuromuscular adaptations, the purpose of this study was to compare the LIF between CMJ and ACMJ while adopting soft and stiff landing strategies. Thirteen resistance-trained athletes (sex: female = 5, male = 8, 26.4 ± 3.7 years, 68.4 ± 13.6 kg, 167 ± 5.1 cm) performed three CMJ and ACMJ each at 60%, 70%, 80% and 90% of bodyweight with instructions to either land soft or stiff on a force plate. Repetitions were separated by 30 s and conditions by 3 min. Resistance bands were used to induce the required weight during ACMJ. Data obtained regarding the average of the two closest trials based on jump height was analysed. Jump height significantly increased with increasing assistance during ACMJ for both landing conditions (*p* < 0.001). Propulsion duration (PD) was significantly shorter with increasing assistance during ACMJ for both landing conditions (*p* < 0.001). Peak and mean propulsion force significantly decreased with increasing assistance during ACMJ for both landing conditions (*p* < 0.001 and *p* < 0.001, respectively). The LIF was significantly greater with increasing assistance during ACMJ in the stiff-landing condition only (*p* < 0.001). Greater assistance allowed participants to jump higher while reducing PD. The higher LIF observed during stiff landing with greater assistance during ACMJ could be attributed to greater jump height and downward velocity during landing.

## 1. Introduction

The countermovement jump (CMJ) is commonly used to assess the neuromuscular capability of the lower limb [1]. In addition, it is also often included in strength training programmes to enhance the various lower limb strength qualities [2,3,4,5,6,7,8,9]. While CMJ has been reported to be effective in improving lower limb neuromuscular capability, researchers have reported that variations in CMJ, such as assisted and resisted CMJ, resulted in greater improvement in jump height [2,8,9,10].

The assisted CMJ (ACMJ) involves attaching a resistance band to the individual, resulting in an unweighting effect which further leads to a form of overspeed training [2,8,9,10,11,12,13]. Due to the unweighting effect, the ACMJ resulted in increased jump height (JH) and take-off velocity with decreased time to take-off (TTO) and propulsion force during ACMJ as compared to CMJ [2,8,9,11,12,14]. Another variable that has been compared between ACMJ and CMJ was the landing impact force (LIF) [2,11,12,13,14]. Tran et al. [11] suggested that chronic exposure to high LIF during training increases athletes’ rate of force development.

Current findings on LIF during ACMJ in comparison to CMJ have been conflicting. For example, Tran et al. [11] and Tufano et al. [13] reported that impact force increased linearly with increased assistance during ACMJ. Tran et al. [11] attributed the increase in LIF during ACMJ to greater jump height, which led to an increase in downward velocity from landing from a greater height. Contrary to these findings, other studies have reported that ACMJ resulted in lower LIF [2,14,15]. This was possibly due to the unweighting effect of the attached resistance band. These conflicting findings could be due to methodological differences, such as differences in populations studied and the resistance band used during ACMJ.

Another possible reason for the difference in LIF observed could be due to different landing strategies of the participants in each study, as a stiff landing would result in greater PLIF than a soft landing technique [16,17]. As none of the studies that investigated the kinetic and kinematics of ACMJ reported on the landing strategy used, it can be assumed that individual participant adopted their own preferred landing strategy. Hence, it is still unknown how much of a difference the LIF would be between ACMJ and CMJ when different landing strategies are adopted.

Knowing if different landing strategies would result in greater LIF during ACMJ in comparison to CMJ may have practical implications for athletes’ training adaptation. Apart from greater LIF, stiff landing has also been reported to result in greater landing impulse of the hip and knee extensor and ankle plantar flexor [16]. Furthermore, greater negative power was also observed for the hip, knee and ankle as drop height increased [17]. Hence, chronic exposure to high LIF during training may benefit athletes who are required to perform activities such as multiple changes in directions and jumps within short periods of time due to greater improvement in the rate of force development [11]. Therefore, the primary purpose of the study was to compare the LIF during ACMJ and CMJ while adopting different landing strategies. The secondary purpose was to compare the jump height (JH) and force–time characteristics of CMJ and ACMJ at different levels of assistance. It was hypothesised that (1) there would be no difference in LIF between ACMJ intensity when adopting a soft landing strategy, while LIF would be increasingly higher with greater assistance during ACMJ when adopting a stiff landing strategy; (2) increased assistance during ACMJ would increase JH but decrease the force–time characteristics metrics values.

## 2. Results

### 2.1. Reliability and Assumptions Check

The reliability data for all measured variables across the different jump intensities are displayed in Table 1. JH at all intensities showed good to excellent reliability (ICC: 0.94–0.98, 95% CI: 0.85–0.99 and %CV: 2.4–3.6). Good reliability was observed for TTO at 60% to 90% (ICC: 0.90–0.93, 95% CI: 0.75–0.97 and %CV: 3.0–3.3), while moderate reliability was observed at 100% (ICC:0.81, 95% CI: 0.57–0.93 and %CV: 5.5). PD at all intensities showed good to excellent reliability (ICC: 0.90–0.97, 95% CI: 0.76–0.99, %CV: 2.5–6.9). Moderate reliability was observed for BD at 80, 90 and 100% (ICC: 0.82–0.84, 95% CI: 0.57–0.94 and %CV: 6.9–8.7) but was unacceptable at 60 and 70% (ICC: 0.69–0.72, 95% CI: 0.37–0.89 and %CV: 11.3–12.3). Unacceptable reliability across all intensities was also observed for UD (ICC: 0.61–0.79, 95% CI: 0.22–0.92 and %CV: 12.1–20.7). MPF showed good to excellent reliability across all intensities (ICC: 0.92–0.99, 95% CI: 0.79–1.00 and %CV: 2.3–9.9). At 90 and 100% intensity, PPF showed excellent reliability (ICC: 0.97–0.99, 95% CI: 0.92–0.99 and %CV: 2.8–4.2) while moderate reliability was observed at the lower intensities of 60, 70 and 80% (ICC: 0.79–0.87, 95% CI: 0.60–0.95 and %CV: 9.1–10.5). Good to excellent reliability was observed for both soft and stiff LIF at 70, 80, 90 and 100% intensities (ICC: 0.88–0.97, 95% CI: 0.75–0.99 and %CV: 7.5–9.8), but was unacceptable at 60% intensity (ICC: 0.81–0.87, 95% CI: 0.68–0.92 and %CV: 10.2–10.6).

Mauchly’s test showed no violation of sphericity for JH (soft: *p* = 0.709, stiff: *p* = 0.889), LIF (soft: *p* = 0.278, stiff: *p* = 0.709), PPF (soft: *p* = 0.110, stiff: *p* = 0.254), TTO (soft: *p* = 0.209, stiff: *p* = 0.632), BD (soft: *p* = 0.120, stiff: *p* = 0.074) and UD (stiff: *p* = 0.537). However, violations of sphericity were observed in MPF (soft: *p* < 0.001, stiff: *p* < 0.001), PD (soft: *p* < 0.001, stiff: *p* < 0.001) and UD (soft: *p* = 0.003). Greenhouse–Geisser adjustment was performed for variables where violations of sphericity were observed.

### 2.2. Jump Height

**Figure 1:**
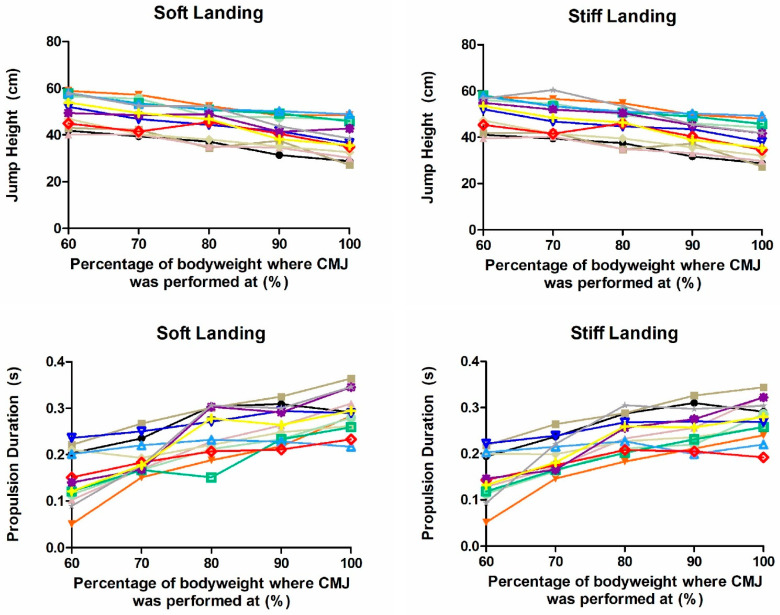
Individual jump height and propulsion duration. Each colour represents a different participant.

Jump height during soft and stiff landing conditions was significantly decreased as intensity increased (F = 63.530, *p* < 0.001; F = 79.225, *p* < 0.001, respectively) (Table 2 and Table 3) (Figure 1). Post hoc analysis revealed that JH for 60_soft_ was significantly higher than all the other intensities (*p* < 0.001, 95% CI = 1.8–15.0, *g* = 0.46–1.69, 95% CI = −0.33–2.59); JH for 70_soft_ was significantly higher than 80_soft_, 90_soft_ and 100_soft_ (*p* < 0.001–0.009, 95% CI = 0.8–11.5, *g* = 0.41–1.31, 95% CI = −0.36–2.17); JH for 80_soft_ was significantly higher than 90_soft_ and 100_soft_ (*p* < 0.001–0.004, 95% CI = 1.4–8.9, *g* = 0.53–0.92, 95% CI = −0.25–1.73); and JH for 90_soft_ was significantly higher than 100_soft_ (*p* = 0.002, 95% CI = 1.5–5.1, *g* = 0.46, 95% CI = −0.32–1.24). The JH for 60_stiff_ was significantly higher than all the other intensities (*p* < 0.001–0.008, 95% CI = 0.75–14.31, *g* = 0.34–1.70, 95% CI = −0.44–2.59); JH for 70_stiff_ was significantly higher than 80_stiff_, 90_stiff_ and 100_stiff_ (*p* < 0.001–0.005, 95% CI = 0.10–12.4, *g* = 0.39–1.37, 95% CI = −0.39–2.22); JH for 80_stiff_ was significantly higher than 90_stiff_ and 100_stiff_ (*p* < 0.001, 95% CI = 1.9–9.2, *g* = 0.53–1.01, 95% CI = −0.24–1.82); and JH for 90_stiff_ was significantly higher than 100_stiff_ (*p* < 0.001, 95% CI = 2.5–5.2, *g* = 0.53, 95% CI = −0.25–1.31).

### 2.3. Temporal Phase Metrics

Time to take-off during soft and stiff landing conditions remained unchanged across intensities (F = 0.156, *p* = 0.959, F = 0.242, *p* = 0.913, respectively) (Table 2 and Table 3). However, PD during soft and stiff landing conditions were significantly affected by intensity (F = 36.253, *p* < 0.001, F = 38.281, *p* < 0.001) (Table 2 and Table 3) (Figure 1). Post hoc analysis revealed that PD for 60_soft_ was significantly shorter than all the other intensities (*p* < 0.001, 95% CI = −0.19–−0.03, *g* = −2.64–−0.86, 95% CI = −3.69–−0.05); PD for 70_soft_ was significantly shorter than 80_soft_, 90_soft_ and 100_soft_ (*p* < 0.001–0.001, 95% CI = −0.13–−0.03, *g* = −2.39–−1.13, 95% CI = −3.31–−0.30); PD for 80_soft_ was significantly shorter than 90_soft_ and 100_soft_ (*p* = 0.001–0.038, 95% CI = −0.07–−0.01, *g* = −0.90–−0.37, 95% CI = −1.71–−0.10); and PD for 90_soft_ was significantly shorter than 100_soft_ (*p* = 0.003, 95% CI = −0.04–−0.01, *g* = −0.67, 95% CI = −1.43–−0.14). The PD for 60_stiff_ was significantly shorter than all the other intensities (*p* < 0.001, 95% CI = −0.17–−0.02, *g* = −2.51–−0.95, 95% CI = −3.54–−0.14); PD for 70_stiff_ was significantly shorter than 80_stiff_, 90_stiff_ and 100_stiff_ (*p* < 0.001–0.001, 95% CI = −0.11–−0.03, *g* = −1.93–−1.21, 95% CI = −2.86–−0.38); PD for 80_stiff_ was significantly shorter than 100_stiff_ (*p* = 0.005, 95% CI = −0.05–−0.01, *g* = −0.79, 95% CI = −1.59–0.01); and PD for 90_stiff_ was significantly shorter than 100_stiff_ (*p* = 0.009, 95% CI = −0.04–−0.01, *g* = −0.51, 95% CI = −1.29–0.22).

Jump intensity also has a significant effect on BD during soft and stiff landing conditions (F = 7.466, *p* < 0.001; F = 12.520, *p* < 0.001, respectively) (Table 2 and Table 3). Significantly longer BD was observed during 60_soft_ than all other intensities (*p* < 0.001–0.036, 95% CI = 0.00–0.12, *g* = 0.43–1.11, 95% CI = −0.35–1.94). The BD for 70_soft_ and 90_soft_ were significantly longer than 100soft (*p* = 0.041, 95% CI = 0.00–0.10, *g* = 0.67, 95% CI = −0.12–1.46, *p* = 0.021, 95% CI = 0.01–0.05, *g* = 0.37, 95% CI = −0.41–1.15,respectively). Similarly, significantly longer BD was observed during 60_stiff_ than all other intensities (*p* < 0.001–0.016, 95% CI = 0.01–0.16, *g* = 0.62–1.13, 95% CI = −0.19–1.96). The BD for 70_stiff_ was significantly longer than 100_stiff_ (*p* = 0.007, 95% CI = 0.02–0.11, *g* = 0.20, 95% CI = −0.57–0.97). The BD for 80_stiff_ and 90_stiff_ were significantly shorter than 100_stiff_ (*p* = 0.015, 95% CI = 0.01–0.05, *g* = −0.34, 95% CI = −1.11–0.44, *p* = 0.008, 95% CI = 0.01–0.15, *g* = −0.27, 95% CI = −1.04–0.50, respectively).

The unweighting duration during soft landing conditions varied significantly across intensities (F = 4.620, *p* = 0.020) but showed no difference during stiff landing conditions (F = 1.792, *p* = 0.146) (Table 2 and Table 3). The UD at 60_soft_, 70_soft_ and 80_soft_ were significantly longer than 90_soft_ and 100_soft_ (*p* = 0.009–0.021, 95% CI = 0.01–0.09, *g* = 0.93–1.00, 95% CI = −0.12–1.82, *p* = 0.006–0.009, 95% CI = 0.01–0.07, *g* = 0.84–0.92, 95% CI = 0.04–1.73, *p* = 0.020–0.021, 95% CI = 0.01–0.08, *g* = 0.78–0.84, 95% CI = −0.02–1.64, respectively).

### 2.4. Force Metrics

**Figure 2:**
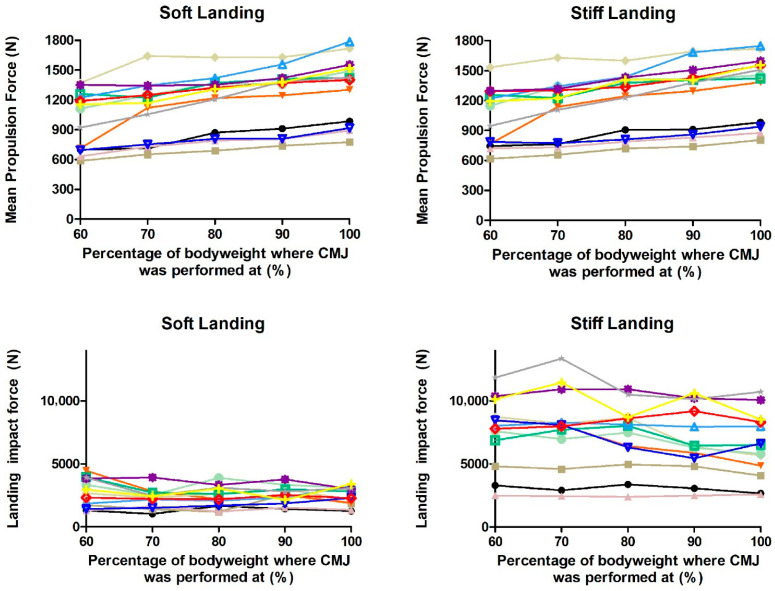
Individual mean propulsion force and landing impact force. Each colour represents a different participant.

Mean propulsion force during soft and stiff landing conditions increased significantly with intensity (F = 19.811, *p* < 0.001; F = 35.223, *p* < 0.001, respectively) (Table 2 and Table 3) (Figure 2). Significantly lower MPF was observed for 60_soft_ than all the other intensities except 70_soft_ (*p* < 0.001–0.004, 95% CI = −525.4–−94.7, *g* = −1.21–−0.79, 95% CI = −2.05–0.01); MPF for 70_soft_ was significantly lower than 80_soft_, 90_soft_ and 100_soft_ (*p* < 0.001, 95% CI = −290.2–−51.8, *g* = −0.70–0.27, 95% CI = −1.49–0.50); MPF for 80_soft_ was significantly lower than 90_soft_ and 100_soft_ (*p* < 0.001–0.005, 95% CI = −192.9–−18.1, *g* = −0.43–−0.16, 95% CI = −1.21–0.61); and MPF for 90_soft_ was significantly lower than 100_soft_ (*p* < 0.01 95% CI = −125.3–−54.4, *g* = −0.28, 95% CI = −1.05–0.50). Significantly lower MPF was observed for 60_stiff_ than all the other intensities except 70_stiff_ (*p* < 0.001–0.025, 95% CI = −407.8–−12.0, *g* = −0.97–−0.30, 95% CI = −1.78–0.48); MPF for 70_stiff_ was significantly lower than 80_stiff_, 90_stiff_ and 100_stiff_ (*p* < 0.001, 95% CI = −292.3–−53.2, *g* = −0.71–−0.09, 95% CI = −1.50–0.68); MPF for 80_stiff_ was significantly lower than 90_stiff_ and 100_stiff_ (*p* < 0.001–0.005, 95% CI = −193.0–−23.8, *g* = −0.50–−0.31, 95% CI = −1.19–0.65); and MPF for 90_stiff_ was significantly lower than 100_stiff_ (*p* < 0.01 95% CI = −101.0–−52.0, *g* = −0.23, 95% CI = −1.06–0.49).

Peak propulsion force during soft and stiff landing conditions was also significantly higher with increasing intensity (F = 22.234, *p* < 0.001; F = 24.681, *p* < 0.001, respectively) (Table 2 and Table 3). Significantly lower PPF was observed for 60_soft_ than all the other intensities except 70_soft_ (*p* < 0.001–0.002, 95% CI = −356.9–−64.1, *g* = −0.80–−0.42, 95% CI = −1.59–0.35); PPF for 70_soft_ was significantly lower than 80_soft_, 90_soft_ and 100_soft_ (*p* < 0.001–0.022, 95% CI = −306.5–−17.3, *g* = −0.70–−0.32, 95% CI = −1.50–0.46); PPF for 80_soft_ and 90_soft_ were significantly lower than 100_soft_ (*p* < 0.001, 95% CI = −190.3–−73.7, *g* = −0.39, 95% CI = −1.17–0.39, *p* < 0.01 95% CI = −152.2–−85.6, *g* = −0.35, 95% CI = −1.12–0.42, respectively). Significant difference in PPF during stiff landing condition was also observed between intensities (F = 24.681, *p* < 0.001, *g* = 0.09–0.78). Significantly lower PPF was observed for 60_stiff_ than all the other intensities except 70_stiff_ (*p* < 0.001–0.005, 95% CI = −350.8–−46.0, *g* = −0.78–−0.39, 95% CI = −1.57–0.39); PPF for 70_stiff_ was significantly lower than 80_stiff_, 90_stiff_ and 100_stiff_ (*p* < 0.001–0.003, 95% CI = −302.0–−38.5, *g* = −0.69–−0.30, 95% CI = −1.48–0.47); PPF for 80_stiff_ and 90_stiff_ were significantly lower than 100_stiff_ (*p* < 0.001, 95% CI = −195.4–−82.8, *g* = −0.42, 95% CI = −1.19–0.36, *p* < 0.001 95% CI = −143.9–−55.0, *g* = −0.28, 95% CI = −1.06–0.49, respectively).

**Figure 3:**
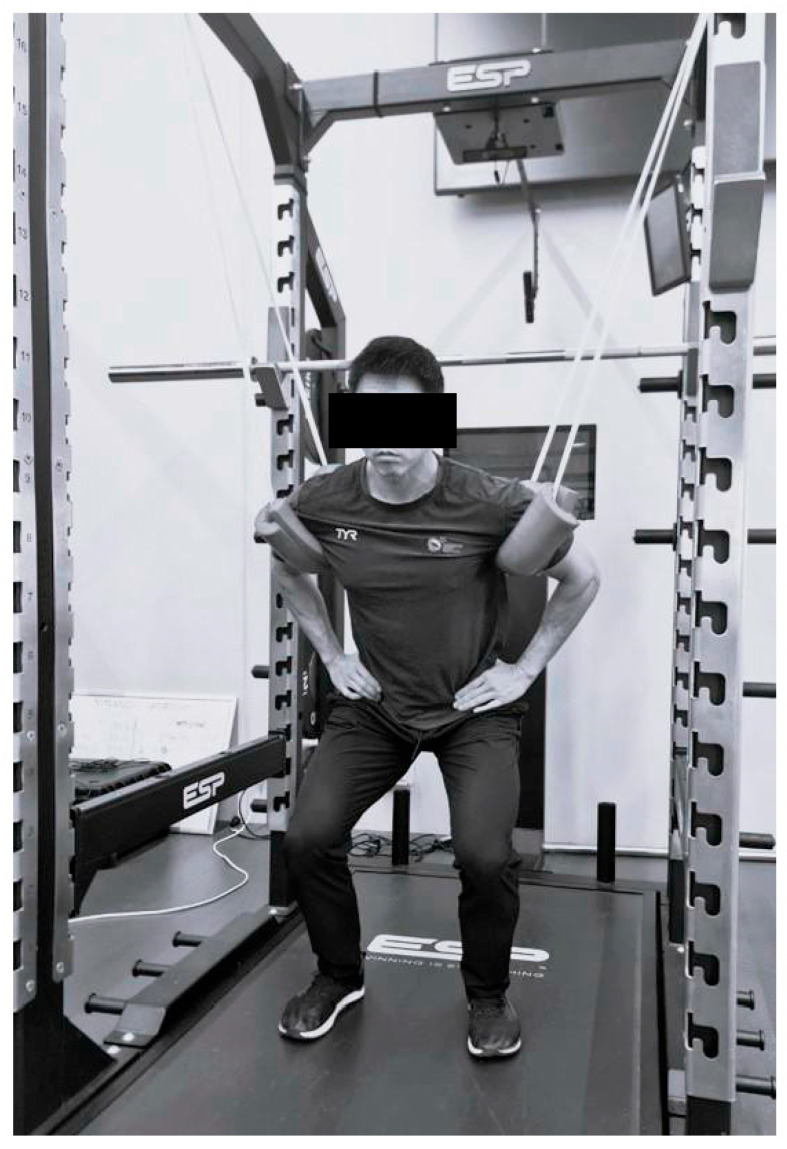
Set up for assisted countermovement jump.

Landing impact force during soft landing conditions did not show a significant change across intensities (F = 1.609, *p* = 0.187) (Table 2 and Figure 3). But LIF during stiff landing conditions were significantly increased with reducing intensity (F = 6.052, *p* < 0.001) (Table 2). The LIF during 60_stiff_, 70_stiff_ and 80_stiff_ were significantly higher than 100_stiff_ (*p* = 0.007, 95% CI = 374.0–1857.4, *g* = 0.41, 95% CI = −0.37–1.19, *p* = 0.002, 95% CI = 553.3–2005.6, *g* = 0.43, 95% CI = −0.35–1.21, *p* = 0.013, 95% CI = 199.3–1355.6, *g* = 0.29, 95% CI = −0.48–1.07, respectively). The LIF during 70_stiff_ was also significantly higher than 90_stiff_ (*p* = 0.021, 95% CI = 171.1–1696.9, *g* = 0.31, 95% CI = −0.46–1.09).

## 3. Discussion

The purpose of the study was to compare the effects of different landing strategies on LIF during CMJ and ACMJ. The current findings showed that stiff landing resulted in increasingly greater LIF as the assistance level for ACMJ increased. However, there was no significant difference in LIF between CMJ and ACMJ across the different levels of assistance when adopting soft landing. Hence, our hypotheses were supported. In addition to the findings of the main objective, JH and BD also exhibited significant increases, while PD, MPF and PPF exhibited significant decreases with greater assistance during ACMJ in both stiff and soft landing conditions. In contrast, UD demonstrated a significant increase with greater assistance during ACMJ only in soft landing.

The results from previous studies comparing the LIF of ACMJ and CMJ reported inconsistent findings [2,5,6,7,8,9,10,11]. The current findings indicated that a possible reason could be due to the lack of standardisation in the landing strategy adopted in those studies. The significantly higher LIF observed in the stiff landing condition as the assistance level increased was likely due to greater downward velocity as a result of a greater jump height. In addition, the resistance provided by the resistance band was possibly not high enough to significantly slow down the downward velocity. However, as movement velocity from the peak jump height to ground contact was not measured, further study will be required to better understand the cause of the greater stiff landing LIF resulting from greater assistance during ACMJ. Nevertheless, it has been suggested that chronic exposure to high LIF during training can provide the stimulus that may lead to the improvement in rate of force development [11]. Hence, based on the current findings, practitioners may include stiff landing in the ACMJ training to enhance adaptations for the rate of force development. It should be noted that creating high LIF by using a landing strategy involving more extended joints may redirect force to passive tissues such as ligaments instead of the musculotendinous unit [18] and may increase the risk of injury. It is, therefore, important to execute the proper stiff landing technique to optimise neuroadaptations and minimise injury risk.

In contrast to the LIF findings for stiff landing, no significant difference in LIF between CMJ and ACMJ across intensities was observed during soft landing, despite the greater jump height observed as during stiff landing, when there was greater assistance for ACMJ. This indicated that during soft landing, participants adopted a landing mechanics that allowed them to accept the LIF over an extended duration. Thus, while the LIF may not differ, the resultant internal load on the musculotendinous system may still be higher for the ACMJ with a greater assistance level [19]. Stiff landing has also been shown to result in greater joint moment and negative power generated [16,17]. Furthermore, it was reported that muscular work at each joint differed between both landing strategies [16]. Devita and Skelly [16] reported that the contribution to the muscular work parameters from the hip, knee and joint were 25, 37 and 37%, respectively, during soft landing, and 20, 31 and 50%, respectively, during stiff landing. The authors also reported greater work being carried out when performing a soft landing due to the greater deceleration distance. This suggests that performing stiff and soft landings during training may result in different adaptations. For example, a stiff landing may acutely increase mechanical loading that could, if applied longitudinally and safely, influence the rate of force development adaptations, while a soft landing may have a beneficial effect on strength adaptations due to greater work involved. In addition, due to the greater distribution of work to the ankle joint as reported by Devita and Skelly [16], stiff landing may provide a greater stimulus for adaptations to muscles around the ankle joint.

The current findings indicate that jump height increased significantly with greater assistance, regardless of the landing strategy used. This observation was consistent with findings from previous studies [2,12]. The increasing jump height observed with the increasing assistance during ACMJ was concurrent with decreased MPF and PPF, and decreased PD. Indicating a higher jump height despite a lower impulse generated. This observation was also consistent with that reported by previous researchers [2,8,13]. This trend could be attributed to the progressive increment in assistance from resistance bands, which provided greater offloading at lower intensities (60% BW) and minimal to no assistance at higher intensities (90–100% BW). Moreover, the level of assistance for each intensity was measured when participants were standing upright. The resultant level of assistance from the resistance band prior to propulsion would have been greater because during the unweighting phase, the resistance bands would have been stretched to a longer length, hence resulting in greater resistance. This would have resulted in participants requiring to overcome less inertia prior to take-off, which led to the significant reduction in MPF and PPF observed.

The current findings also showed a lack of significant difference in total TTO across intensities. This was not concurrent with previous findings [8,11]. The lack of significant changes in TTO in this study could suggest that participants were able to adjust phase-specific timing while maintaining overall jump execution efficiency, which highlights their adaptability of neuromuscular coordination in managing force production and transition mechanics across the different phases before take-off. Future studies may include joint kinematic or electromyography analysis to better understand the muscle-tendon behaviour during CMJ and ACMJ.

As the assistance level decreased, PD became progressively longer, suggesting that jumping with greater mass requires a prolonged force application during the propulsion. The longer PD concurrent with higher MPF resulted in a higher impulse that allows individuals to overcome the inertia to lift off the ground during the CMJ. However, in the case of ACMJ, the resistance band reduced the amount of inertia that participants were required to overcome. This led to a shorter amount of time taken to generate the required amount of force to propel themselves off the force plate. Researchers of previous studies have also reported similar findings [2,8,12]. In addition, studies using ACMJ as a training intervention have attributed the reduced PD as a form of overspeed stimulus that enhanced the neuromuscular adaptation from ACMJ in comparison to CMJ [2,8,10].

No previous studies performed have measured the two temporal variables of BD and UD during ACMJs. The current findings showed that BD was significantly affected by intensity, with longer durations observed at lower intensities compared to higher intensities. It could be possible that participants were attempting to overcome any constraints caused by the resistance bands in the process of achieving their optimal preferred countermovement depth during the ACMJ [20]. UD was significantly influenced by intensity, particularly in soft landing conditions. It is worthwhile to note here that UD had unacceptable reliability across intensities, and possible inter- and intra-individual variability could have resulted in the above observations. The increased UD and BD might have been the reason for the lack of change in TTO despite a significant decrease in PD.

Readers should take into consideration several limitations while interpreting the current results. Firstly, the foot landing technique (forefoot or flatfoot) was not specified to the participants. A study by Dario and David [21] investigated neuromuscular characteristics of drop jumps and hurdle jumps using different landing techniques, showing that forefoot landing resulted in greater ground reaction force and stiffness as well as a shorter ground contact time than flatfoot landing. Hence, this factor could possibly have an impact on the current results. Future studies may consider controlling this factor. Secondly, the current protocol required participants to reset their position after each jump instead of performing the jump continuously like a rebound jump. Hence, the findings may differ if participants were to perform the CMJ and ACMJ using the rebound jump methods. Thirdly, due to the small sample size and specific athletes from each individual sport, the result may not be generalised for other demographics. In addition, the a priori power calculated for this sample size was based on one variable, while the study measured multiple variables. Fourthly, the current results may be specific to the resistance bands used in this study and may yield different findings if other types of resistance bands were to be used. Finally, the current results only provide possible insights into the type of adaptations that may occur while performing the two different landing methods; an intervention study is required in order to elucidate the neuromuscular adaptations that may occur.

## 4. Materials and Methods

### 4.1. Study Design

This study adopted a repeated-measure counterbalance design. Participants were required to attend one familiarisation and one testing session 48–96 h apart. During each session, participants completed a single set of three CMJs with 90%, 80%, 70% and 60% bodyweight with either stiff (100_stiff_, 90_stiff_, 80_stiff_, 70_stiff_ and 60_stiff_, respectively) or soft (100_soft_, 90_soft_, 80_soft_, 70_soft_ and 60_soft_, respectively) landing. Each set was performed in a randomised order with three minutes of recovery to avoid an order effect or any potentiation effect.

### 4.2. Sample

A priori power analysis with an effect size of 0.4, power of 90%, and an α error of 0.05 was conducted, and the estimated sample size was 13 participants. Hence, 5 resistance-trained female (age: 25.0 ± 3.6 years, body mass: 54.1 ± 7.6 kg and height: 160.2 ± 3.8 cm) and 8 male (age: 27.8 ± 3.9 years, body mass: 77.2 ± 8.1 kg and height: 174.9 ± 6.4 cm) national level athletes from cycling (*n* = 1), handball (*n* = 1), kayaking (*n* = 3), powerlifting (*n* = 1), rowing (*n* = 1), squash (*n* = 1) and track sprinting (*n* = 5) were recruited for this study. To be included in this study, participants must be (1) 18–45 years old; (2) have been performing resistance and plyometric training for at least 1 year; and (3) have not sustained any chronic illness or injury 6 months prior to participation. Participants have been participating in their respective sports competitively for >3 years, performing between 10 and 12 h of sports training and 4–8 h of strength training per week. They have also been performing the CMJ regularly as part of training and testing during their career as national athletes. All testing commenced after obtaining approval from the Institutional Review Board of Singapore Sport Institute (SC-EXP−038). Written informed consent was obtained from the participants for the publication of any potentially identifiable images or data included in this article.

### 4.3. Procedures

The standardised warm-up performed during each session included 5 min of self-paced moderate intensity cycling on a cycling ergometer. Subsequently, participants performed 10 repetitions of body weight squat, hip hinge, forward lunge, submaximal CMJ and ankle hops. A 1 min recovery period was provided prior to performing the main activity of each session.

During the familiarisation session, the resistance level of the resistance band (Domyos elastic training band), requiring the induction of 90%, 80%, 70% and 60% of participants’ bodyweight, was established. The average percentage body mass reduced by the resistance bands for the 90%, 80%, 70% and 60% conditions were 10.8 (1.7)%, 18.7 (1.8)%, 28.1 (2.4)% and 37.5 (3.5)%, respectively. This was accomplished by having participants stand on a dual-force plate (Force Decks, VALD Performance, FD4000, Newstead, Queensland, Australia), sampling at 1000 Hz. The position of the force plate relative to the squat rack, where resistance bands were attached, was fixed throughout the experimental period. Markings were drawn on the force plate to position participants in order to maintain consistent tension for the resistance band. Resistance bands of different thicknesses were attached to participants by placing them under the armpits of the participants (Figure 3). A sponge padding was positioned between the participants’ armpits and the resistance bands to prevent abrasion. Weight measurement was performed with participants standing upright with their knee and hip joints fully extended. Subsequently, participants were asked to perform three jumps at each intensity with either a stiff or soft landing in a random order. Each repetition was separated by 30 s and each condition by 3 min. Participants returned 48–96 h later for the actual testing session, where they performed the same procedure.

The CMJ was performed with participants keeping their arms akimbo to prevent any arm swing. During the ACMJ, the resistance bands required to induce the specific intensity were attached to a horizontal traverse bar in the gym. Participants looped bands around their arms with the band in contact with the armpits (Figure 3). Participants were instructed to maintain the position of their hands throughout the execution of the ACMJ. The stiff and soft landings were defined as knee angles > 90° and <90°, respectively [16]. All trials were monitored visually to ensure that the correct landing technique was performed with both feet completely on the force plate. For the stiff landing condition, participants were instructed to land stiffly to produce a loud “bang” upon landing and to stop any further knee and hip flexion as quickly as possible. Participants were also instructed not to land with extended knees and hips to avoid injury. For the soft landing condition, they were instructed to land softly and accept the force by flexing the knee and hip to a greater degree, and to be as silent as possible during landing. Both CMJ and ACMJ were performed on the same dual force plates. The commercially available ForceDecks software version 2.1.0 (VALD Performance, ForceDecks, Newstead, Queensland, Australia) was used to analyse and generate the jump variables [22]. A 20 N offset from the measured body mass was used to define the initiation of the jump. Hence, participants were required to stand as still as possible for >1 s prior to the execution of the countermovement. The time point at which the total vertical force fell below the threshold of 20 N was used to define the initiation of take-off [23]. Dependent variables included JH, TTO, propulsion duration (PD), braking duration (BD), unweighting duration (UD), mean (MPF) and peak propulsion force (PPF), as well as LIF. The MPF, PPF and LIF were analysed and expressed as absolute force. LIF was defined as the peak ground reaction force during first contact upon landing. The average values for all variables obtained from the two closest trials based on jump height were recorded and analysed. One trial was excluded for each condition.

### 4.4. Data Analysis

All tested variables were expressed by mean (±1 SD) and analysed using SPSS version 29.0. Two-way, mixed intraclass correlation coefficients (ICCs) [ICC (3,1)] and a coefficient of variation (%CV) for all measured variables were used to assess within-session test–retest reliability. ICC values were considered poor, moderate, good, or excellent if the lower-bound 95% confidence interval (CI) of ICC values was <0.50, 0.50–0.74, 0.75–0.90, or >0.90, respectively [24]. Acceptable within-session variability was classified as <10% [25]. All assumptions to run ANOVA were checked using Mauchly’s test. A repeated measures analysis of variance (ANOVA) with Bonferroni post hoc analysis was used to assess if any between-intensities difference exists for all variables within each landing condition. Hedge’s *g* was calculated as a standardised effect size for mean comparisons, and deemed as (i) a trivial effect size if *g* < 0.25; (ii) a small effect size if *g* = 0.25–0.50; (iii) a moderate effect size if *g* = 0.50–1.0; and (iv) a large effect size if *g* > 1.0 [26].

## 5. Conclusions

Assisted countermovement jump (ACMJ) offers a different training stimulus that may result in different neuromuscular adaptations based on the level of assistance and landing strategies as compared to CMJ. When performed with a stiff landing, ACMJs may acutely increase mechanical loading that could influence the rate of force development adaptations. The limited joint flexion during landing requires rapid force production, which could be ideal for developing the explosive strength needed in actions like sprinting or quick directional changes. However, it should be noted that high LIF may also increase the risk of injury (19). Hence, it is important to maintain a low training volume when implementing stiff landing and to ensure proper landing mechanics. There should also be careful monitoring of musculoskeletal soreness or pain to prevent injuries. In contrast, using soft landing increases joint motion and time under tension that may lead to higher work being performed. This may promote improvements in eccentric strength and hypertrophy. Additionally, performing ACMJ with a shorter propulsion duration can provide an overspeed training effect that may result in enhanced neuromuscular adaptations as reported in earlier studies. By adjusting these variables, ACMJs can be purposefully integrated into training programmes to support a range of athletic development objectives.

## Figures and Tables

**Table 1:** Reliability data.

Percentage Bodyweight	Variable	ICC (95% CI)	Qualitative Rating	%CV (95% CI)
100	CMJ Height (cm)	0.97 (0.92–0.99)	Excellent	3.6 (2.7–5.6)
	Time to Take-Off (ms)	0.81 (0.57–0.93)	Moderate	5.5 (4.1–8.5)
	Propulsion Duration (s)	0.91 (0.78–0.97)	Good	4.7 (3.5–7.2)
	Braking Duration (s)	0.83 (0.60–0.93)	Moderate	8.7 (6.5–13.4)
	Unweighting Duration (s)	0.67 (0.31–0.86)	Poor	12.1 (9.0–18.9)
	Mean Propulsion Force (N)	0.99 (0.98–1.00)	Excellent	2.3 (1.8–3.5)
	Peak Propulsion Force (N)	0.97 (0.92–0.99)	Excellent	4.2 (3.2–6.4)
	Soft Landing Impact Force (N)	0.96 (0.89–0.98)	Good	7.5 (5.6–11.5)
	Stiff Landing Impact Force (N)	0.97 (0.93–0.99)	Excellent	7.8 (5.9–12.1)
90	CMJ Height (cm)	0.97 (0.93–0.99)	Excellent	2.6 (1.9–3.9)
	Time to Take-Off (s)	0.93 (0.82–0.97)	Good	3.0 (2.3–4.6)
	Propulsion Duration (s)	0.97 (0.93–0.99)	Excellent	2.5 (1.8–3.7)
	Braking Duration (s)	0.84 (0.62–0.94)	Moderate	7.9 (5.9–12.3)
	Unweighting Duration (ms)	0.76 (0.46–0.90)	Poor	13.3 (9.9–20.9)
	Mean Propulsion Force (N)	0.99 (0.98–1.00)	Excellent	2.5 (1.8–3.7)
	Peak Propulsion Force (N)	0.99 (0.96–0.99)	Excellent	2.8 (2.1–4.3)
	Soft Landing Impact Force (N)	0.94 (0.86–0.98)	Good	7.9 (5.9–12.3)
	Stiff Landing Impact Force (N)	0.97 (0.92–0.99)	Excellent	8.7 (6.5–13.5)
80	CMJ Height (cm)	0.98 (0.94–0.99)	Excellent	2.4 (1.8–3.7)
	Time to Take-Off (s)	0.93 (0.81–0.97)	Good	3.2 (2.4–4.9)
	Propulsion Duration (s)	0.97 (0.93–0.99)	Excellent	3.0 (2.3–4.6)
	Braking Duration (s)	0.82 (0.57–0.93)	Moderate	6.9 (5.2–10.7)
	Unweighting Duration (ms)	0.61 (0.22–0.84)	Poor	20.7 (15.3–33.0)
	Mean Propulsion Force (N)	0.92 (0.79–0.97)	Good	8.4 (6.3–13.0)
	Peak Propulsion Force (N)	0.87 (0.69–0.95)	Moderate	9.1 (6.8–14.1)
	Soft Landing Impact Force (N)	0.92 (0.80–0.97)	Good	8.1 (6.1–12.4)
	Stiff Landing Impact Force (N)	0.97 (0.91–0.99)	Excellent	8.9 (6.6–13.7)
70	CMJ Height (cm)	0.94 (0.85–0.98)	Excellent	3.4 (2.5–5.1)
	Time to Take-Off (ms)	0.92 (0.79–0.97)	Good	3.0 (2.2–4.5)
	Propulsion Duration (s)	0.92 (0.79–0.97)	Good	5.5 (4.1–8.4)
	Braking Duration (s)	0.72 (0.39–0.89)	Poor	11.3 (8.4–17.6)
	Unweighting Duration (s)	0.79 (0.53–0.92)	Moderate	14.8 (11.0–23.3)
	Mean Propulsion Force (N)	0.99 (0.98–1.00)	Excellent	9.4 (7.1–14.2)
	Peak Propulsion Force (N)	0.81 (0.63–0.92)	Moderate	9.1 (6.8–14.1)
	Soft Landing Impact Force (N)	0.88 (0.75–0.93)	Good	9.5 (7.2–13.8)
	Stiff Landing Impact Force (N)	0.93 (0.86–0.97)	Good	9.8 (7.5–14.8)
60	CMJ Height (cm)	0.95 (0.87–0.98)	Good	3.2 (2.4–4.9)
	Time to Take-Off (s)	0.90 (0.75–0.96)	Good	3.3 (2.4–5.0)
	Propulsion Duration (s)	0.90 (0.76–0.96)	Good	6.9 (4.8–9.4)
	Braking Duration (s)	0.69 (0.37–0.87)	Poor	12.3 (9.3–18.5)
	Unweighting Duration (ms)	0.72 (0.46–0.85)	Poor	16.8 (12.5–25.0)
	Mean Propulsion Force (N)	0.92 (0.89–0.95)	Excellent	9.9 (7.5–15.0)
	Peak Propulsion Force (N)	0.79 (0.60–0.89)	Moderate	10.5 (7.3–15.3)
	Soft Landing Impact Force (N)	0.81 (0.68–0.91)	Moderate	10.2 (7.8–14.9)
	Stiff Landing Impact Force (N)	0.87 (0.80–0.92)	Good	10.6 (7.5–15.4)

**Table 2:** Performance measures of all variables during soft landing condition.

Variables	Intensity						
	60%	70%	80%	90%	100%	F-Value	*p*
CMJ Height (cm)	50.9 (6.9) ^aabbccdd^	47.8 (6.4) ^bbccdd^	45.0 (6.6) ^ccdd^	41.5 (6.1) ^dd^	38.2 (7.629)	63.530	<0.001
Time to Take-off (s)	0.818 (0.077)	0.819 (0.082)	0.830 (0.096)	0.822 (0.089)	0.822 (0.096)	0.156	0.959
Propulsion Duration (s)	0.151 (0.058) ^aabbccdd^	0.194 (0.037) ^bbccdd^	0.246 (0.051) ^cdd^	0.263 (0.038) ^dd^	0.290 (0.043)	36.253	<0.001
Braking Duration (s)	0.447 (0.075) ^abbccdd^	0.414 (0.074) ^d^	0.372 (0.074)	0.391 (0.068) ^d^	0.365 (0.068)	7.466	<0.001
Unweighting Duration (s)	0.220 (0.066) ^cdd^	0.211 (0.058) ^ccdd^	0.211 (0.065 ^cd^	0.168 (0.039)	0.167 (0.030)	4.620	0.003
Mean Propulsion Force (N)	993.1 (292.2.0) ^bbccdd^	1095.2 (301.4) ^bbccdd^	1181.0 (305.4 ^ccdd^	1231.9 (305.4) ^dd^	1321.7 (326.5)	19.811	<0.001
Peak Propulsion Force (N)	1319.5 (320.0) ^bbccdd^	1356.4 (303.5) ^bccdd^	1458.3 (315.9) ^dd^	1471.4 (318.0) ^dd^	1590.3 (339.3)	22.234	<0.001
Landing Impact Force (N)	2696.5 (1129.4)	2248.3 (745.1)	2371.6 (824.7)	2523.0 (679.0)	2440.1 (713.7)	1.609	0.187

^aa^ Denotes significant difference from 70% intensity (*p* < 0.01); ^b^ Denotes significant difference from 80% intensity (*p* < 0.05); ^bb^ Denotes significant difference from 80% intensity (*p* < 0.01); ^c^ Denotes significant difference from 90% intensity (*p* < 0.05); ^cc^ Denotes significant difference from 90% intensity (*p* < 0.01); ^d^ Denotes significant difference from 100% intensity (*p* < 0.05); ^dd^ Denotes significant difference from 100% intensity (*p* < 0.01).

**Table 3:** Performance measures of all variables during stiff landing condition.

Variables	Intensity						
	60%	70%	80%	90%	100%	F-Value	*p*
CMJ Height (cm)	50.9 (7.0) ^aabbccdd^	48.5 (7.1) ^bbccdd^	45.7 (6.9) ^ccdd^	42.0 (6.3) ^dd^	38.2 (7.5)	79.225	<0.001
Time to Take-off (s)	0.795 (0.068)	0.791 (0.082)	0.800 (0.085)	0.788 (0.087)	0.801 (0.098)	0.242	0.913
Propulsion Duration (s)	0.151 (0.053) ^aabbccdd^	0.196 (0.037) ^bbccdd^	0.243 (0.038) ^cdd^	0.254 (0.041) ^dd^	0.276 (0.043)	38.281	<0.001
Braking Duration (s)	0.451 (0.087) ^aabbccdd^	0.402 (0.070) ^dd^	0.367 (0.053) ^dd^	0.369 (0.069) ^dd^	0.388 (0.067)	12.520	<0.001
Unweighting Duration (s)	0.193 (0.040)	0.193 (0.054)	0.190 (0.039)	0.166 (0.028)	0.187 (0.034)	1.792	0.146
Mean Propulsion Force (N)	1040.6 (289.5) ^abbccdd^	1119.8 (298.0) ^bbccdd^	1152.6 (430.8) ^ccdd^	1273.7 (325.8) ^dd^	1350.0 (329.9)	35.223	<0.001
Peak Propulsion Force (N)	1346.1 (316.7) ^bbccdd^	1374.8 (318.8) ^ccdd^	1471.2 (305.7) ^dd^	1510.8 (339.3) ^dd^	1610.3 (342.3)	24.687	<0.001
Landing Impact Force (N)	7608.2 (2709.3) ^dd^	7771.8 (3137.6) ^cdd^	7269.9 (2530.9) ^d^	6837.8 (2645.9)	6492.4 (2583.7)	6.052	<0.001

^a^ Denotes significant difference from 70% intensity (*p* < 0.05); ^aa^ Denotes significant difference from 70% intensity (*p* < 0.01); ^bb^ Denotes significant difference from 80% intensity (*p* < 0.01); ^c^ Denotes significant difference from 90% intensity (*p* < 0.05); ^cc^ Denotes significant difference from 90% intensity (*p* < 0.01); ^d^ Denotes significant difference from 100% intensity (*p* < 0.05); ^dd^ Denotes significant difference from 100% intensity (*p* < 0.01).

## Data Availability

The raw data supporting the conclusions of this article will be made available by the authors on request.

## Abbreviations

The following abbreviations are used in this manuscript:

60_soft_	Assisted countermovement jump at 60% body weight with soft landing
60_stiff_	Assisted countermovement jump at 60% body weight with stiff landing
70_soft_	Assisted countermovement jump at 70% body weight with soft landing
70_stiff_	Assisted countermovement jump at 70% body weight with stiff landing
80_soft_	Assisted countermovement jump at 80% body weight with soft landing
80_stiff_	Assisted countermovement jump at 80% body weight with stiff landing
90_soft_	Assisted countermovement jump at 90% body weight with soft landing
90_stiff_	Assisted countermovement jump at 90% body weight with stiff landing
100_soft_	Countermovement jump with soft landing
100_stiff_	Countermovement jump with stiff landing
ACMJ	Assisted countermovement jump
BD	Braking duration
CMJ	Countermovement jump
LIF	Landing impact force
MPF	Mean propulsion force
PPF	Peak propulsion force
PD	Propulsion duration
TTO	Time to take-off
UD	Unweighting duration

## References

[1] Bishop C., Turner A., Jordan M., Harry J., Loturco I., Lake J., Comfort P. (2022). A framework to guide practitioners for selecting metrics during the countermovement and drop jump tests. Strength Cond. J..

[2] Argus C.K., Gill N.D., Keogh J.W., Blazevich A.J., Hopkins W.G. (2011). Kinetic and training comparisons between assisted, resisted, and free countermovement jumps. J. Strength Cond. Res..

[3] Gehri D.J., Ricard M.D., Kleiner D.M., Kirkendall D.T. (1998). A comparison of plyometric training techniques for improving vertical jump ability and energy production. J. Strength Cond. Res..

[4] James L.P., Talpey S.W., Young W.B., Geneau M., Newton R.U., Gastin P.B. (2023). Strength classification and diagnosis: Not all strength is created equal. Strength Cond. J..

[5] Makaruk H., Starzak M., Suchecki B., Czaplicki M., Stojiljković N. (2020). The effects of assisted and resisted plyometric training programs on vertical jump performance in adults: A systematic review and meta-analysis. J. Sports Sci. Med..

[6] Markovic G. (2007). Does plyometric training improve vertical jump height? A meta-analytical review. Br. J. Sports Med..

[7] Mirzaei B., Norasteh A.A., Asadi A. (2013). Neuromuscular adaptations to plyometric training: Depth jump vs. countermovement jump on sand. Sport Sci. Health.

[8] Sheppard J.M., Dingley A.A., Janssen I., Spratford W., Chapman D.W., Newton R.U. (2011). The effect of assisted jumping on vertical jump height in high-performance volleyball players. J. Sci. Med. Sport.

[9] Strate M., Stien N., Saeterbakken A.H., Andersen V. (2022). The effects of assisted and resisted plyometric training on jump height and sprint performance among physically active females. Eur. J. Sport Sci..

[10] Markovic G., Vuk S., Jaric S. (2011). Effects of jump training with negative versus positive loading on jumping mechanics. Int. J. Sports Med..

[11] Tran T.T., Brown L.E., Coburn J.W., Lynn S.K., Dabbs N.C., Schick M.K., Schick E.E., Khamoui A.V., Uribe N.P., Noffal G.J. (2011). Effects of different elastic cord assistance levels on vertical jump. J. Strength Cond. Res..

[12] Tran T.T., Brown L.E., Coburn J.W., Lynn S.K., Dabbs N.C. (2012). Effects of assisted jumping on vertical jump parameters. Curr. Sports Med. Rep..

[13] Tufano J.J., Malecek J., Steffl M., Stastny P., Hojka V., Vetrovsky T. (2018). Field-based and lab-based assisted jumping: Unveiling the testing and training implications. Front. Physiol..

[14] Markovic G., Jaric S. (2007). Positive and negative loading and mechanical output in maximum vertical jumping. Med. Sci. Sports Exerc..

[15] Tufano J.J., Vetrovsky T., Stastny P., Staffl M., Malecek J., Omcirk D. (2022). Assisted jumping in healthy older adults: Optimizing high-velocity training prescription. J. Strength Cond. Res..

[16] Devita P., Skelly W.A. (1992). Effect of landing stiffness on joint kinetics and energetics in the lower extremity. Med. Sci. Sports Exerc..

[17] Verniba D., Vescovi J.D., Hood D.A., Gage W.H. (2017). The analysis of knee joint loading during drop landing from different heights and under different instruction sets in healthy males. Sports Med.-Open.

[18] Pedley J.S., Lloyd R.S., Read P.J., Moore I.S., Myer G.D., Oliver J.L. (2025). Drop jump vertical kinetics identify male youth soccer players at greater risk of non-contact knee injury. Phys. Ther. Sport.

[19] Mills C., Pain M.T., Yeadon M.R. (2009). Reducing ground reaction forces in gymnastics’ landings may increase internal loading. J. Biomech..

[20] Malachy P.M., Cohen J.A., Karl F.O., Kremenic I.J. (2024). Effect of countermovement depth on the neuromechanics of a vertical jump. Transl. Sports Med..

[21] Dario F.C., David G.B. (2013). Neuromuscular characteristics of drop and hurdle jumps with different types of landings. J. Strength Cond. Res..

[22] Linthorne N.P. (2001). Analysis of standing vertical jumps using a force platform. Am. J. Phys..

[23] Heishman A., Daub B., Miller R., Brown B., Freitas E., Bemben M. (2019). Countermovement jump inter-limb asymmetries in collegiate basketball players. Sports.

[24] Koo T.K., Li M.Y. (2016). A guideline of selecting and reporting intraclass correlation coefficients for reliability research. J. Chiropr. Med..

[25] Cormack S.J., Newton R.U., McGuigan M.R., Doyle T.L.A. (2008). Reliability of measures obtained during single and repeated countermovement jumps. Int. J. Sports Physiol. Perform..

[26] Flanagan E.P. (2013). The effect size statistic—Applications for the strength and conditioning coach. Strength Cond. J..

